# Threat induction biases processing of emotional expressions

**DOI:** 10.3389/fpsyg.2022.967800

**Published:** 2022-11-24

**Authors:** Aleya Flechsenhar, Seth Levine, Katja Bertsch

**Affiliations:** ^1^Clinical Psychology and Psychotherapy, Department of Psychology, LMU Munich, Munich, Germany; ^2^NeuroImaging Core Unit Munich (NICUM), University Hospital LMU, Munich, Germany; ^3^Department of General Psychiatry, Center for Psychosocial Medicine, Heidelberg University, Heidelberg, Germany

**Keywords:** attention, context dependency, emotion recognition, eye tracking, face processing, induced threat, psychophysics

## Abstract

Threats can derive from our physical or social surroundings and bias the way we perceive and interpret a given situation. They can be signaled by peers through facial expressions, as expressed anger or fear can represent the source of perceived threat. The current study seeks to investigate enhanced attentional state and defensive reflexes associated with contextual threat induced through aversive sounds presented in an emotion recognition paradigm. In a sample of 120 healthy participants, response and gaze behavior revealed differences in perceiving emotional facial expressions between threat and safety conditions: Responses were slower under threat and less accurate. Happy and neutral facial expressions were classified correctly more often in a safety context and misclassified more often as fearful under threat. This unidirectional misclassification suggests that threat applies a negative filter to the perception of neutral and positive information. Eye movements were initiated later under threat, but fixation changes were more frequent and dwell times shorter compared to a safety context. These findings demonstrate that such experimental paradigms are capable of providing insight into how context alters emotion processing at cognitive, physiological, and behavioral levels. Such alterations may derive from evolutionary adaptations necessary for biasing cognitive processing to survive disadvantageous situations. This perspective sets up new testable hypotheses regarding how such levels of explanation may be dysfunctional in patient populations.

## Introduction

An alley can feel threatening on our way home alone at night, much more than it would during the day. An embarrassment weighs stronger with an audience than in private. These examples indicate that context shapes the way humans act and react in certain situations and during interactions with one another. For some, the feeling of threat is ubiquitous as soon as others are present. The fear of a negative response to an immediate threat or aversive experience, such as being judged or embarrassed, is a core symptom of individuals with social anxiety (World Health Organization, 2020). This leads to heightened sensitivity for social information, as reported by a number of studies investigating threat processing in these populations, expressed in the form of slower approach reactions to threat (Heuer et al., 2007; Roelofs et al., 2009), a higher attentional focus toward threatening facial expressions (Lazarov et al., 2016; Gregory et al., 2018), as well as faster responses to threatening information, such as important social threat cues like fearful or angry facial expressions (Asmundson and Stein, 1994).

A general sensitivity to threat is evident for healthy populations as well, seeing as it is evolutionarily plausible and adaptive to detect unknown dangers in the environment (Pourtois, 2004; Bublatzky et al., 2020). This threat bias has been found consistently in emotion research (see meta-analysis of Lisk et al., 2020). Immersed in a threatening context, healthy individuals have also shown reduced visual exploration and heart rate and an overall increase in electrodermal activity when given the opportunity for flight (Rösler and Gamer, 2019), stronger negativity biases (Müller-Pinzler et al., 2019), and different interpretations of emotions (Bublatzky et al., 2020; Kavcıoğlu et al., 2021). Prior studies also suggest that anticipation can influence face perception on the electrocortical (Bublatzky et al., 2014) and behavioral levels (Kavcıoğlu et al., 2021). Such converging evidence supports the modulatory role or contextual settings on perception in general and on the recognition of facial emotions (Wieser and Brosch, 2012).

Specifically, Bublatzky et al. (2020) found that ambiguous facial expressions presented during an instructed threat-of-shock led to a biased recognition of fearful, but not happy facial expressions. Furthermore, contextual threat enhanced fear processing when facial expressions were difficult to categorize (e.g., ambiguous morphs or ambiguous emotions, such as surprise; Neta et al., 2017), while safety enhanced processing of subtle happy faces. The authors conclude that contextual settings reduce the salience threshold and reveal a congruency effect in boosting early face processing of low-expressive emotions, while incongruent face-contexts drive neural activity of easier recognizable emotions.

The threat-of-shock paradigm is an experimental procedure, in which participants are verbally informed about the fact that they may receive electric shocks during the presentation of a specific cue, while another cue indicates a safety period, where shocks never occur (Bublatzky et al., 2014). The Threat-of-shock paradigm has been shown to selectively guide attention, activate the autonomic nervous system, prime defensive reflexes, and trigger behavioral avoidance (Paret and Bublatzky, 2019), rendering it an efficient tool to “model” the perceptions of individuals with generalized anxiety. These paradigms allow for the investigation of behavioral and cognitive changes relevant for (mal)adaptive anxiety (Robinson et al., 2013b; Bublatzky et al., 2014).

Compared to induced threat, certain facial expressions themselves indicate social threat. While expressions of anger of one person signal direct threat to the perceiver, fearful expressions indicate potential threat in the environment (Kavcıoğlu et al., 2021). Generally speaking, emotional expressions have perceptual priority over neutral expressions (Alpers and Gerdes, 2007), and elevated state anxiety can enhance this priority (Gray, 2009). With regard to threat deriving from the environment, one should keep in mind that social cues are always embedded in some situational context in real life, be it through eye gaze, facial dynamics, affective prosody, body postures, or verbal descriptions that signal different variations of threat (Wieser and Brosch, 2012). For example, a neutral face in a work context indicates no concern, while a neutral face during a conversation with a friend may be interpreted negatively. The smile of a stranger is potentially more unexpected than the smile of a family member upon meeting them. However, specifically changing the situational context experimentally yields an opportunity to systematically investigate contextual influences on face processing. Previous studies applying threat-of-shock have utilized electric shocks as aversive context induction, yet acoustic information serves as a natural context induction that can bias the identification of a facial emotion in the direction of the simultaneously presented affective prosody, serving as a bimodal emotion perception (de Gelder and Vroomen, 2000). Although researchers have investigated the impact of aversive anticipation on visual perception of emotional facial expressions (see review of Robinson et al., 2013b), few have examined precise attentional influences using eye tracking in a threat-of-shock paradigm that includes acoustic threat. This setup has a number of advantages: First, reflexive and sustained attentional processes can be differentiated as a function of context during the processing of emotional facial expressions. Distinguishing between reflexive (automatic eye movements measured for short stimulus presentation times of below 200 ms) and sustained attention (voluntary eye movements over longer stimulus presentation times) is an advantage in capturing differences in bottom-up and top-down processes. Socially anxious individuals for example, have previously shown a pronounced tendency to reflexively attend to emotional facial expressions, yet subsequently avoid them (e.g., Boll et al., 2016). This hypervigilance could not be measured with long presentation times. Second, replication as well as differences in behavior between contexts induced with electric shocks in previous studies can be examined using a bimodal emotion perception, in the form of visual perception paired with auditory cues. Through this procedure, we are able to induce more socially relevant threats, in the form of screams, gunshots, or panic as opposed to electric shocks that are unrelated to social situations or interactions. Third, simultaneous behavioral responses, *via* key press, allow for the analysis of performance in recognizing emotions in different contexts in parallel to eye movement data, thereby overcoming limitations of previously applied Stroop or dot-probe tasks to measure attention allocation.

The aim of this study was to investigate three questions: (1) How does contextual threat influence emotion recognition? (2) Are the contextual differences stronger for specific emotions? (3) Are there differences in personality traits and person-related variables that influence differences in behavior between contexts? Previous evidence pertaining to the recognition of faces under threat is divergent, with some studies reporting improved perceptual processing of highly expressive fearful faces (Kavcıoğlu et al., 2021), while others indicate negative impacts of threat (Bosse and Schnitfink, 2015). We hypothesized that, on a behavioral level, reaction times would be slower and error rates higher for facial expressions presented in a threatening context, due to higher arousal interfering with cognitive processes for classifying emotions and promotion of response inhibition (see Robinson et al., 2013a). With regard to gaze behavior, we anticipated longer dwell times (fixations) as suggested by “freezing behavior” (Rösler and Gamer, 2019) and faster initial orienting (saccades) to facial expressions in the threat context in line with the literature on hypervigilance (Boll et al., 2016) induced by the context manipulation. We expected contextual differences to also be stronger for emotions signaling social threat, such as anger and fear. We suspected that the expectation of threat or aversion may be analogous to the perceptual experiences of particularly socially anxious individuals, considering the aversive sounds and emotional facial expressions of our setup. Hence, we anticipated that more (socially) anxious individuals would show smaller behavioral differences between contexts as compared to less anxious individuals. To this end, we collected data on social anxiety, as well as general state and trait anxiety. Previous studies not only found attentional biases to threat in clinical and dispositional anxiety, but also revealed qualitatively different types of threat biases, such as preferential engagement, difficulty in disengagement, or attentional avoidance (Cisler and Koster, 2010; Sheppes et al., 2013; but see Robinson et al., 2013b). Furthermore, autistic, or antisocial tendencies have been found to elicit reduced emotion recognition abilities and altered gaze patterns (e.g., Black et al., 2020; Kimonis et al., 2020). To account for such traits, we investigated Spearman’s correlations between behavioral and eye movement measures and these trait variables. We also included a respective questionnaire to ensure that results were not influenced by negative or positive affect as positive mood has been associated with more global gaze behavior, while negative mood relates to local processing (Schmid and Schmid Mast, 2010) and decreased performance in emotion recognition, as well as negative bias (Schmid et al., 2011).

## Materials and methods

### Participants

The study was approved by the ethics committee of the Department of Psychology at the Ludwig-Maximilians-Universität in Munich and conducted according to the principles expressed by the Declaration of Helsinki following its 2013 revision. All subjects gave written informed consent and received payment or course credit as reimbursement for their time participating. A total of 144 participants were recruited and tested for the study comprising two separate experiments. Two participants were excluded due to prior diagnoses of Depression and Borderline Personality Disorder. Twenty-two participants were excluded due to missing or insufficient (<70% clean) eye tracking data, or (<30%) reaction time data, which is comparable with previous eye tracking studies (Flechsenhar and Gamer, 2017; Flechsenhar et al., 2018b). Two different setups were applied to test reflexive and sustained attentional mechanisms by using a brief (150 ms) and longer (5,000 ms) presentation time for presenting stimuli tested on different participants that were randomly assigned. Participants were recruited and tested until a final number of 120 participants (*n* = 60 for each experiment; see *a priori* power-analysis below) was reached. Ten participants were of non-German origin (specifically, Croatian, Persian, French, Turkish, Malaysian, and Spanish); 53 participants were male and 67 female. Inclusion criteria as a prerequisite to participate in the study were as follows: age between 18 and 45 years, no color blindness, no wearing of glasses (participation was allowed if subjects could switch to soft contact lenses), no prior diagnosis of mental illness, and a proficiency level in German of at least B2 according to the Common European Framework of Reference for Languages.

### Questionnaires

To assess social anxiety, we included the Liebowitz Social Anxiety Scale (LSAS; Stangier and Heidenreich, 2005), which is a self-report measure, consisting of 24 items depicting different social situations that are rated on a 4-point Likert scale. It is further divided into two subscales for fear and avoidances. Heimberg et al. (1999) and Baker et al. (2002) evaluated the psychometric properties of the LSAS and found high internal consistency (α = 0.95) and high convergent validity with other measures of social anxiety. In our sample, the internal consistency yielded α = 0.94. The scoring scale differentiates between moderate social phobia with sum scores between 55 and 65, marked social phobia between 65 and 80, and severe social phobia with a score of over 80 points.

The German version of the State/Trait Anxiety Inventory (STAI; Laux et al., 1981) was used to assess current and habitual fear and to differentiate further between fear as a state or trait variable. It consists of 20 items evaluated on a 4-point scale, which are summed up to a total score ranging from 20 to 80 points. Internal consistency ranges from α = 0.88–0.94 on the trait scale and α = 0.90–0.94 on the state scale. In our sample, the internal consistency for the STAI-S yielded α = 0.82 and for the STAI-T α = 0.90.

The German short version of the Autism-Spectrum Quotient (AQ-K; Freitag et al., 2007) was used to assess the degree of traits associated with the autistic spectrum. The questionnaire consists of 33 items, which assess three different areas (social interaction and spontaneity, imagination and creativity, communication and reciprocity) rated on a 4-point scale. A cutoff value is set at a score of above 17 points. Alpha coefficients range from α =0.65 to 0.87 reflecting a moderate to high internal consistency. In our sample, the internal consistency yielded α = 0.69.

The Levenson Self-Report Psychopathy Scale (LSRP translated to German; Levenson et al., 1995) represents a widely used self-report questionnaire that employs a 4-point Likert scale for assessing psychopathic traits in non-forensic populations (Lilienfeld and Fowler, 2006) and has been used to measure antisocial dispositions. It consists of 26 items rated on a 4-point scale and has a moderate to high internal consistency (see Hicklin and Widiger, 2005; α = 0.66; Lynam et al., 1999; α = 0.68; Miller et al., 2001; α = 0.63, and Ross et al., 2004; α = 0.62). The LSRP is divided in two positively correlated subscales: Primary Psychopathy (LSRP–PP; 16 items, α = 0.82) and Secondary Psychopathy (LSRP–SP; 10 items, α = 0.63). The primary psychopathy subscale reflects interpersonal and affective features including manipulation, egocentricity, a lack of empathy, and a lack of remorse, whereas the secondary psychopathy subscale assesses social deviance behaviors such as impulsivity, stimulation seeking, and poor behavioral control (Osumi and Ohira, 2017). Concerning cutoff values, 0–48 classifies as “non-psychopathic,” 49–57 as “mixed group,” and above 58 as “psychopathic group” (Brinkley et al., 2001). In our sample, the internal consistency yielded α = 0.71.

The German version of the Positive Affect Negative Affect Schedule (PANAS; Watson et al., 1988; Janke and Glöckner-Rist, 2014) was used to control for extreme effects of mood on the behavioral and eye movement data. Negative affect subsumes feelings of unhappiness and aversion, while positive affect includes joy, which are measures in 20 items with 5 answer options. Internal consistency is high with α = 0.85–0.89 (Crawford and Henry, 2004). In our sample, the internal consistency yielded α = 0.76.

Spearman’s correlations were calculated exploratorily between all psychometric, behavioral, and eye movement measures using difference values between safety and threat contexts. We therefore did not correct for multiple testing.

### Stimulus material

Sounds played during the threatening contexts were taken from the International Affective Digital Sounds (IADS; Bradley and Lang, 2007). Images for the training phase were partly taken from the International Affective Picture System (IAPS; Lang et al., 2008), but also from internet searches (e.g., Google and Pixabay). Images of the facial expressions were selected from several picture databases [the Karolinska directed emotional faces, KDEF; (Lundqvist et al., 1998); Pictures of facial affect, NimStim (Tottenham et al., 2009), and the FACES database (Ebner et al., 2010)]. Faces were slightly rotated such that both pupils were always horizontally aligned, then converted to gray-scale images and cropped with an ellipse. Cumulative brightness was normalized across pictures. One hundred and twenty stimuli were presented in the experimental paradigm with the respective emotional expressions anger, fear, happiness, and a neutral expression. Half of the stimuli showed a male face, the other a female face. Fifteen stimuli were presented within one block. Emotional expressions were counterbalanced randomly across context conditions. Each identity was presented four times, once for each emotional expression.

**Figure 1:**
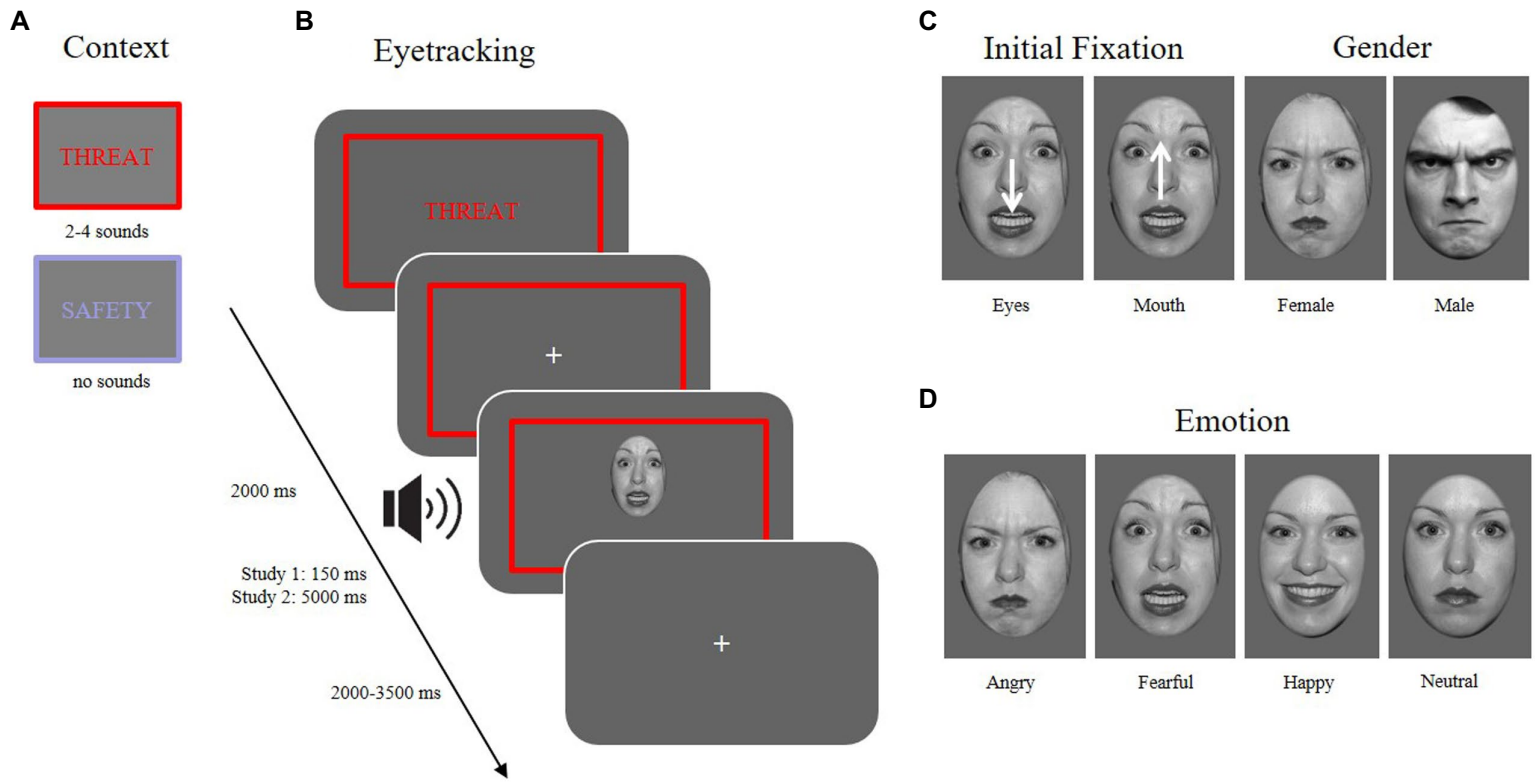
Different conditions of the experimental paradigm. **(A)** *Context*: two possible context manipulations (threat vs. safety). **(B)** *Eyetracking*: an example trial sequence in the threaten context with an aversive sound playing at the onset of a facial expression (~13–27% contingency) while measuring eye movements. **(C)** *Initial fixation*: the face is presented either with the mouth at the location of the previously attended fixation cross, or the face was shifted downward, so that the eyes were presented at the location of the fixation cross. *Gender*: 50% of the presented stimuli were male, 50% female faces. **(D)** *Emotion*: Stimuli used in the experiment (and depicted here) were taken from the NimStim database (Tottenham et al., 2009) and included four emotional expressions (angry, fearful, happy, neutral).

To control for the initial fixation, stimuli within each emotional expression were shifted either downward or upward on each trial, leading to either the eye or the mouth region to appear at the location of the previously presented fixation cross (see Figure 1C).

### Eye tracking

Eye movements were measured with a desktop-mounted EyeLink 1,000 (SR Research Ltd., Ottawa, Canada) with sampling rate of 1,000 Hz and a 25 mm lens. The experiment was programmed with Presentation^©^ (Neurobehavioral Systems Inc., Version 21.1, Berkeley, CA, United States). Stimuli were presented on a 24″ display (Dell Precision 3,630) with a resolution of 1920 × 1,080 pixels. Participants sat at a constant viewing distance of 65 cm from the monitor with their head placed in a chin rest with a forehead band that was mounted to the table. The experiment was conducted in a laboratory room with dim uniform lighting conditions. A 9-point calibration was used to ensure high-quality monocular eye tracking.

### Procedure

There were two experimental setups: one in which the stimuli were shown for a total duration of 150 ms to examine reflexive attentional processes (indicated as ‘Study 1’ in Figure 1) and the other with a duration of 5,000 ms to investigate sustained processing of emotional expressions (indicated as ‘Study 2’). Both setups were conducted in the same room, following the same procedure and stimuli, but performed by different participants, as different attentional mechanisms were of interest for this study, namely reflexive and sustained visual orientation. Previous studies have shown differences in emotion processing with regard to this differentiation (e.g., Scheller et al., 2012; Flechsenhar et al., 2018a).

Participants filled out the Liebowitz Social Anxiety Scale (LSAS; Stangier and Heidenreich, 2005) questionnaire online prior to the appointment in the laboratory. Upon arrival, participants were given instructions, and filled out the STAI-State questionnaire *via* paper-pencil. Then, they were seated in front of the computer and asked to place their chin on the chinrest and wear headphones. Each session consisted of three phases: (1) adjustment, (2) training, and (3) main experiment.

1. In the *adjustment phase*, participants were shown 12 pictures, of which 6 depicted neutral non-social situations in the form of animals and plants, and 8 depicted social scenes which showed physical violence or mobbing between two or more individuals. The aversive social stimuli were paired with a red frame (height: 880–900 pixels, width: 1200–1,180 pixels, and RGB-values: 255, 0, 0) and a suitable sound, while the neutral non-social pictures were presented with a blue frame (RGB-values: 128, 128, 255). Participants were also verbally instructed that a red frame indicated that they would receive unpleasant sounds *via* headphones, but that no sounds would be presented in the trials with a blue frame. The adjustment phase was introduced to our paradigm, as temporally preceding information has previously been found to mediate affective priming effects (Hietanen and Leppänen, 2008; Diéguez-Risco et al., 2013, 2015).
2. The *training phase* consisted of two blocks containing 16 trials, 8 trials for the safety condition and 8 for the threat condition in which each of the four emotional expressions was presented twice. Out of 12 different sounds, 2–4 randomly chosen sounds were presented within one block. This frequency was chosen according to previous literature (Neta et al., 2017; Bublatzky et al., 2020) and was kept at a lower frequency to avoid habituation.
3. The *main experiment* consisted of 8 blocks with 15 trials each, resulting in 120 trials. Half of the experimental blocks were presented as a threat condition with instructed threat-of-acoustic-shock, the other half was presented as a safety condition. Blocks alternated across the experiment and the assignment and order of context were counterbalanced across participants, such that odd-numbered participant codes started with the threatening context, while even-numbered codes started with the safety context. A total of 120 facial stimuli were used with the four different emotional expressions (angry, fearful, happy, and neutral) from 15 male faces and 15 female faces. At the beginning of each block, participants were shown the context condition indicating either “THREAT” or “SAFETY” for 5 s in the color of the respective frame. At the beginning of every trial, subjects would see a fixation cross for 2 s, followed by the presentation of a face (either shifted up or downward, again to control for the initial fixation) that, in the threat context, could be combined with a sound that was previously heard in the adjustment phase. Upon stimulus presentation, participants were tasked with responding as quickly and accurately as possible to the emotional expression shown, even if the face was no longer visible onscreen. Responses were given *via* key presses on a keyboard “V,” “B,” “N,” and “M” marked with the emotional expressions “neutral,” “angry,” “happy,” and “fearful.” The key combinations were switched for each experiment after half of the participants were recruited. After 150 or 5,000 ms, respective of the experiment, a fixation cross would be shown for 2000–3,500 ms before the next trial began. Responses were recorded from stimulus onset until the beginning of the next trial and subsequently filtered in data analysis to below 2000 ms for both setups. During the entire trial within one block, a colored frame would be presented according to the context condition.

Due to a longer duration, a short break was given for Study 2 after 60 trials of 120 were completed. Upon starting the second recording, the calibration and validation process was repeated. After completing the experiment, participants filled out the questionnaires (including a second state anxiety measure) on the same computer using the computer mouse to select their responses.

### Data analysis

**Figure 2:**
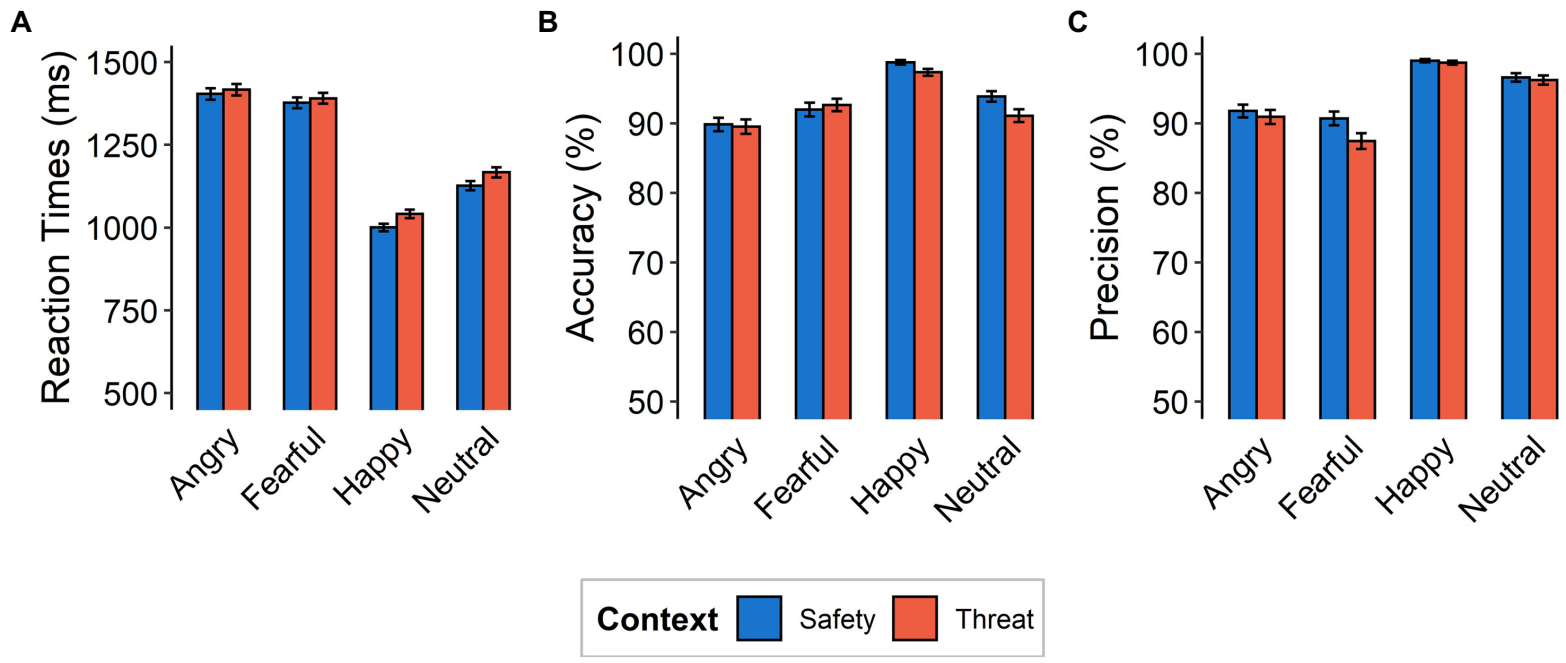
Behavioral Data. **(A)** Mean reaction times (in milliseconds), **(B)** accuracy, and **(C)** precision as a function of emotional expression (angry, fearful, happy, neutral) and context (safety, threat). Note that the y-axes do not begin at the natural zero. Error bars indicate standard errors of the mean.

Reaction times, accuracy, and precision were calculated from the behavioral data for each context, initial fixation, and emotional expression. Reaction times were assessed using only correctly classified responses (Figure 2) and only include response latencies of >200 ms and < 5,000 ms to exclude unintentional premature button-presses or delayed reactions (see Rösler and Gamer, 2019 for comparison). Accuracy was calculated as the number of correct responses for a given condition divided by the number of presentations of that condition [P(“X”|X), i.e., probability of providing a particular answer (e.g., “Angry”), given that the corresponding condition (i.e., Angry) occurred]. Precision was calculated as the number of presentations of a given condition divided by the number of responses of that condition [P(X|“X”), i.e., probability of a particular condition occurring (e.g., Angry), given that the corresponding answer (i.e., “Angry”) was provided]. Reaction times, accuracy, and precision were analyzed using 2 × 4 repeated-measures ANOVAs with factors context (threat and safety) and emotional expression (angry, fearful, happy, and neutral). The setup allows for differentiation between reflexive and sustained attentional processes that were evaluated in separate cohorts in this study. However, results do not aim at comparing these setups but instead focus on influences of aversive anticipation within these mechanisms of attention.

We analyzed the first saccade after stimulus onset (*M* = 409.77 ms, *SD* = 613.16 ms), which occurred 100 ms after stimulus presentation, as Crouzet (2010) found that latencies toward faces occur as early as 100 ms. Eye movements were segmented into saccades and fixations using velocity and acceleration thresholds of 30°/s and 8,000°/s^2^, respectively, for saccade detection. Time intervals between saccades were defined as fixations. Fixations were drift corrected according to a baseline reference period of 300 ms before stimulus onset, during the presentation of a fixation cross. Outliers of baseline coordinates were identified using a recursive outlier removal procedure that was applied separately to x-and y-baseline-coordinates. For each subject, the highest and lowest baseline coordinates were temporarily removed and the mean and standard deviation were calculated for the remaining data. If either of the two values fell outside an interval bounded by 3 standard deviations from the mean, the baseline coordinate was entirely removed. If the data points remained within the interval, they were returned to the data set. Trials with invalid baseline position data were replaced by the means of all valid baseline positions, including a removed x- or y-baseline coordinate or missing baseline data (*M* = 7.19%, *SD* = 6.10%). The latency of the first saccade was measured as the time between the onset of the stimulus and the first fixation (*M* = 409.77 ms, *SD* = 613.16 ms). The proportion of fixation changes reflects the number of saccades made during the presentation of the stimulus, while the dwell time indicates the duration of a fixation.

Due to the setup, saccade data were analyzed for both experiments (*n* = 120), yet fixation data could only be extracted from longer presentation durations (*n* = 60). For the analyses of fixations, we calculated the overall dwell times for each participant, as well as the fixation density of the first three subsequent fixations made after stimulus onset. Fixation densities refer to two-dimensional maps created for each participant and stimulus where fixations are weighted by their durations (milliseconds) and then additively assigned to the map at the pixel position of the fixation. The resulting maps are then smoothed with a two-dimensional isotropic Gaussian kernel with 1° of visual angle using the R package *spatstat* (Baddeley et al., 2015) and the scale of the fixation density maps normalized (see, e.g., Flechsenhar and Gamer, 2017). In addition to the factors context and emotional expression, we included diagnostic features in our analyses. Fixational measures were used to investigate attention distribution onto the two features of the eye region and the mouth region of the faces presented, which represent a diagnostic value in categorizing emotions. That is, the exploration of happy faces has been associated with focusing the mouth rather than the eyes, while fearful and angry faces have been found to draw attention to the eyes (see, e.g., Calvo et al., 2014). Dwell times were analyzed as a function of context (threat and safety), emotional expression (angry, fearful, happy, and neutral), and feature, i.e., the amount of time spent fixating the diagnostic region of the eyes versus the mouth of a presented face. This results in a 2 × 2 × 4 repeated-measures ANOVA (context, feature, and emotional expression). Sequential fixations refer to the trajectory of gaze behavior, focusing on the first three fixations made after stimulus onset. Considering this factor of fixation number, we included the face as a whole (excluding eyes and mouth regions), as an additional feature variable. To grasp the succession of relevant facial features (eyes, mouth, face) across emotional expressions (angry, fearful, happy, and neutral) and context (safety and threat), we analyzed the first three sequential fixations using a 2×3×3×4 repeated-measures ANOVA.

To differentiate between important diagnostic features, as has been done in previous research (e.g., Bertsch et al., 2017), we marked regions of interest (ROI) for the eyes and mouth of each emotional expression. Pixel coordinates were defined for each region, respectively, by manual drawing in GIMP to assign each ROI pixel a certain color (i.e., green and red). Effects of initial fixations are mentioned in the results section, yet do not represent the central feature of this paper. Common effects were replicated from previous research utilizing this paradigm (Scheller et al., 2012; Boll et al., 2016; Seitz et al., 2021). Differences between experiments (study 1 and study 2) regarding brief and longer presentation durations were evident, yet we refrain from extensive elaboration on these results, as the focus of analyses was to investigate contextual effects and both setups could be used to assess initial attention orientation (see Supplementary Table 1 for details).

Data were therefore analyzed across both studies using the open-source statistical programming language R (^1^version 1.2.5019; R Core Team, 2016) and SPSS (version 26). The R-package ez (version 4.4; Lawrence, 2016) was used for all repeated-measures ANOVAs. To assess the influence of social anxiety, we conducted repeated-measures analyses of covariance (ANCOVAs) with the within-subject factors context, initial fixation, and emotional expression. For the other questionnaire data, we used correlational analyses.

We conducted power analyses (Faul et al., 2007) to calculate the number of participants necessary for revealing medium-sized effects in repeated-measures ANOVAs (*f* = 0.25) at a critical alpha level of 0.05 with statistical power of at least 0.80 (Cohen, 1962). Partial η^2^ (Bakeman, 2005) and Cohen’s *d* are reported as estimates of the effect size for ANOVAs and t-tests, respectively. The Huynh–Feldt procedure (Huynh and Feldt, 1976) was used for all repeated-measures ANOVAs containing more than one degree of freedom in the enumerator to account for potential violations of the sphericity assumption (reported as Ɛ). *Post-hoc* tests were paired *t*-tests, to assess the driving factors underlying omnibus tests confirmed by the ANOVA.

## Results

### The influence of contextual threat on emotion recognition

#### Behavioral data

Reaction times for correctly classified emotional expressions were higher for the threat context than for the safety context [*F*_(1,117)_ = 12.77, *p =* 0.0005, ƞ_p_^2^ = 0.10]. Subjects were generally slowest in responding to fearful faces (*M* = 1410.0, *SD* = 12.1 ms), followed by angry ones (*M* = 1384.0, *SD* = 11.6 ms), then neutral expressions (*M* = 1146.0, *SD* = 10.4 ms), while happy faces were categorized the quickest (*M* = 1021.0, *SD* = 8.62 ms), resulting in a main effect of emotional expression [*F*_(3,351)_ = 89.79, *p* < 0.001, ƞ_p_^2^ = 0.43, Ɛ = 0.84; see Figure 2A]. We did not find evidence for an interaction between emotional expressions and context [*F*_(3,351)_ = 0.87, *p =* 0.456, ƞ_p_^2^ = 0.007, Ɛ = 1].

**Figure 3:**
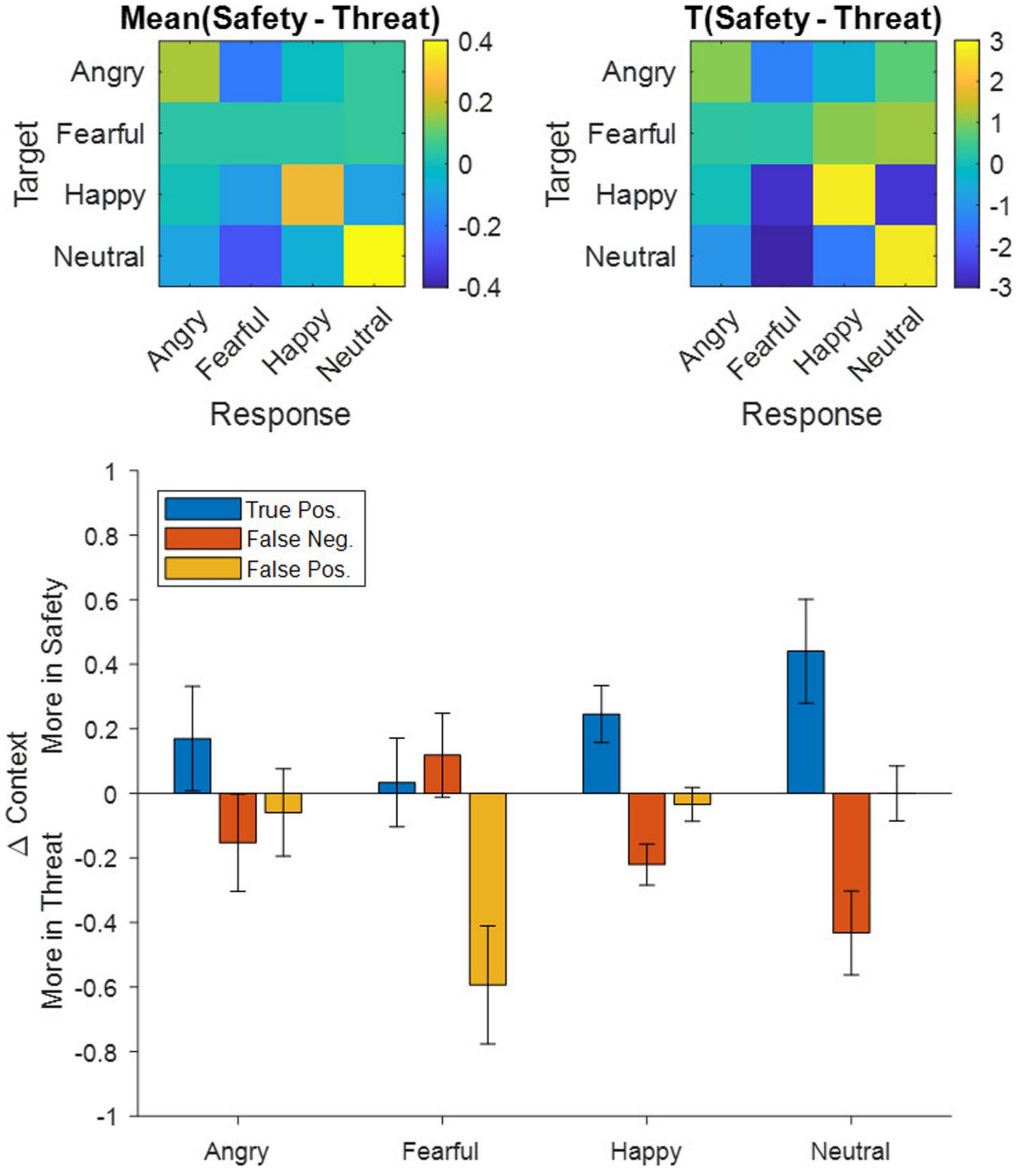
**Top**: Confusion matrices for (**left**) averaged group-level differences between the safety and threat contexts and (**right**) the corresponding t-scores depicting emotional expressions shown in the experiment (target) as rows and the corresponding responses of the participants as columns. Warm colors indicate more responses in the safety context, while cool colors indicate more responses in the threat context. **Bottom**: Breakdown of the group-averaged accuracy and precision results (ref. Figure 2) into their corresponding true positive, false negative, and false positive counts contrasted between contexts (i.e., Safety-Threat) as a function of the four emotional expressions. Error bars indicate standard error of the mean.

Accuracy and precision analyses were calculated for each subject and each condition separately. Accuracy was high overall (*M* = 93.12%, *SD* = 13.18%) and differed between contexts with lower accuracy under threat [*F*_(1,117)_ = 5.42, *p =* 0.02, ƞ_p_^2^ = 0.04]. A main effect of emotional expression [*F*_(3,351)_ = 18.15, *p* < 0.001, ƞ_p_^2^ = 0.14, Ɛ = 0.82] describes higher accuracy for categorizing happy faces (98.0%) and lower accuracy for angry (89.7%), fearful (92.3%), and neutral (92.5%) expressions. Most critically, we found evidence for an interaction between emotional expression and context [*F*_(3,351)_ = 3.07, *p =* 0.03, ƞ_p_^2^ = 0.03, Ɛ = 0.87], driven more by lower accuracy in the threat context (compared to the safety context) for happy [*t*_(117)_ = 3.44, *p* = 0.0008, *d* = 0.32] and neutral [*t*_(117)_ = 3.43, *p* = 0.0008, *d* = 0.32] expressions, than by angry [*t*_(117)_ = 0.91, *p* = 0.365, *d* = 0.08] and fearful [*t*_(117)_ = −0.73, *p* = 0.469, *d* = 0.07] expressions (Figures 2B, 3 Top).

With respect to precision, we also observed a difference between contexts [*F*_(1,117)_ = 7.14, *p* = 0.009, ƞ_p_^2^ = 0.06] and emotions [*F*_(3,351)_ = 61.576, *p* < 0.001, ƞ_p_^2^ = 0.35, Ɛ = 0.87], as well as an interaction effect [*F*_(3,351)_ = 3.85, *p* = 0.01, ƞ_p_^2^ = 0.03, Ɛ = 0.73]. This interaction effect was driven more so by decreased precision under threat (compared to safety) for fearful responses [*t*_(117)_ = 2.83, *p* = 0.005, *d* = 0.26], than for angry [*t*_(117)_ = 1.06, *p* = 0.29, *d* = 0.10], happy [*t*_(117)_ = 0.83, *p* = 0.41, *d* = 0.08], and neutral [*t*_(117)_ = 0.70, *p* = 0.49, *d* = 0.06] responses (Figures 2C, 3 Top).

To supplement the accuracy analysis, we additionally carried out a signal detection analysis (Macmillan and Creelman, 2004) by exploring whether the sensitivity index d’ (which incorporates both the true positive rate and the false positive rate) also differed between contexts and emotions. Ultimately, results were similar between d’ and accuracy, in that d’ values were lower under threat [*F*_(1,117)_ = 8.21, *p =* 0.005, ƞ_p_^2^ = 0.07] and differed across emotional expressions [*F*_(3,351)_ = 98.61, *p* < 0.001, ƞ_p_^2^ = 0.457, Ɛ = 0.70]. However, unlike the accuracy results, we did not observe an interaction between context and emotion for d’ [*F*_(3,351)_ = 0.986, *p* = 0.388, ƞ_p_^2^ = 0.008, Ɛ = 0.83].

To explore whether differences in the accuracy and precision results stemmed from decreases in the true positives (TP) or rather from increases in the false positives (FP) and false negatives (FN), we decomposed all participants’ accuracy (TP/[TP + FN]) and precision (TP/[TP + FP]) scores into their constituent TPs, FNs, and FPs, subtracted them between contexts (i.e., Safety – Threat) and then averaged them across participants (Figure 3 Bottom). Following a Holm–Bonferroni correction, the adjusted threshold was *p* < 0.0156, revealing a greater number of average FP in the threat context for fearful responses [*t*_(117)_ = −3.24, *p* = 0.0016, *d* = 0.30], a greater number of average FN in the threat context for happy [*t*_(117)_ = −3.45, *p* = 0.0008, *d* = 0.32] and neutral [*t*_(117)_ = −3.33, *p* = 0.0012, *d* = 0.31] expressions, and a greater number of average TP in the safety context for happy [*t*_(117)_ = 2.78, *p* = 0.0063, *d* = 0.26] and neutral expressions [*t*_(117)_ = 2.74, *p* = 0.0071, *d* = 0.25]. Remaining effects did not surpass the Holm–Bonferroni threshold [*t*_(117)_ < |1.05|, *p* > 0.2978].

#### Gaze behavior

**Figure 4:**
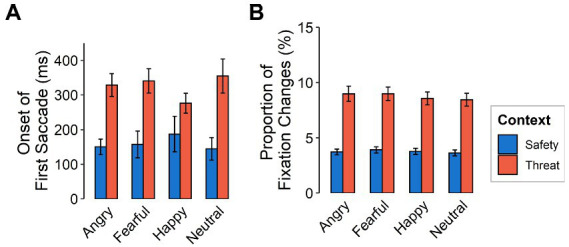
Gaze behavior as a function of emotional expression (angry, fearful, happy, neutral) and context (safety, threat) for **(A)** latency of the first saccade after stimulus onset (in ms) and **(B)** proportion of fixation changes. Error bars indicate standard error of the mean.

The *initiation of the first saccade after stimulus onset* was made later in the threat context than in the safety context [*F*_(1,116)_ = 93.65, *p* < 0.001, ƞ_p_^2^ = 0.45] yet did not differ between emotional expressions [*F*_(3,348)_ = 0.40, *p* = 0.757, ƞ_p_^2^ = 0.003; see Figure 4A]. When considering saccades with initial fixation on the mouth, onsets of the first saccade were more delayed than for initial fixations on the eyes [*F*_(1,116)_ = 9.44, *p* = 0.003, ƞ_p_^2^ = 0.08].

*Fixation changes* were also made more frequently in the threat compared to the safety context [*F*_(1,116)_ = 196.86, *p* < 0.001, ƞ_p_^2^ = 0.63] but did not differ between emotional expressions (see Figure 4B). Fixation changes from to the mouth were higher overall, compared to those to the eyes [*F*_(1,116)_ = 20.97, *p < 0*.001, ƞ_p_^2^ = 0.15]. We did not find evidence for interactions between emotional facial expressions and context [*F*_(3,348)_ = 0.14, *p =* 0.936, ƞ_p_^2^ = 0.001, Ɛ = 0.95], between feature and context [*F*_(1,116)_ = 1.78, *p =* 0.182, ƞ_p_^2^ = 0.02], nor between feature and emotional expression [*F*_(3,348)_ = 1.13, *p =* 0.337, ƞ_p_^2^ = 0.01, Ɛ = 1]. We also found no evidence for a three-way interaction [*F*_(3,348)_ = 0.58, *p =* 0.631, ƞ_p_^2^ = 0.01, Ɛ = 1].

**Figure 5:**
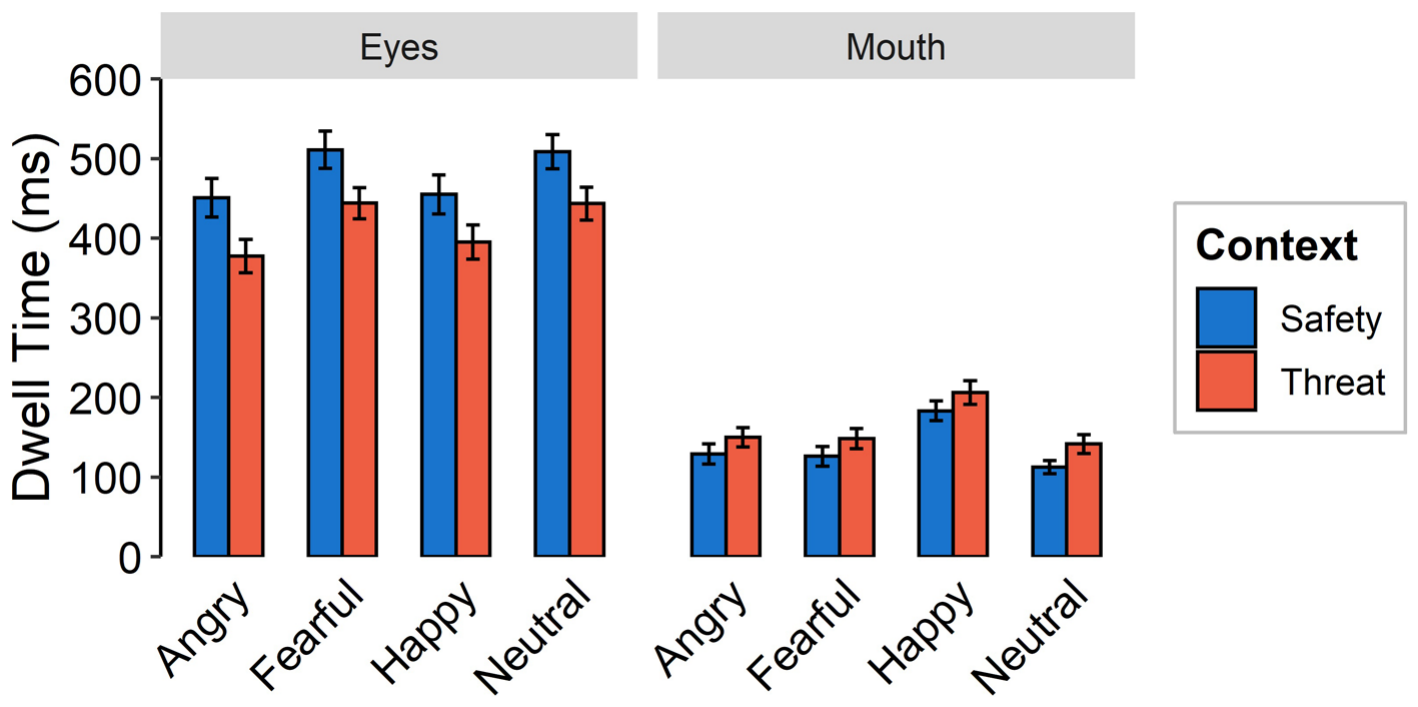
Dwell times as a function of emotional expression (angry, fearful, happy, and neutral) and context (threat, safety) onto the eye or mouth region (feature) of a presented face. Error bars indicate standard error of the mean.

*Dwell times* were shorter under threat than for safety contexts [*F*_(1,59)_ = 23.94, *p <* 0.001, ƞ_p_^2^ = 0.30]. There was a higher focus on the mouth in the threat context across all emotional expressions, while the eye region was focused longer in the safety context across all emotional expressions [interaction between context and feature: *F*_(1,59)_ = 99.03, *p <* 0.001, ƞ_p_^2^ = 0.63; see Figure 5]. Independent of context, the longest dwell times were found for happy faces, followed by fearful and neutral faces, while angry faces were focused least, yielding a main effect of emotional expression [*F*_(3,174)_ = 23.85, *p <* 0.001, ƞ_p_^2^ = 0.29, Ɛ = 0.96]. Dwell times overall were higher for the eyes than for the mouth [*F*_(1,59_ = 116.18, *p <* 0.001, ƞ_p_^2^ = 0.66]. An interaction between emotional expression and feature [*F*_(3,174)_ = 37.05, *p <* 0.001, ƞ_p_^2^ = 0.39, Ɛ = 0.90] indicates longer dwell times on the eyes of fearful and neutral expressions compared to angry and happy faces. Dwell time on the mouth region of happy faces was higher than for all other facial expressions. We did not find evidence for an interaction effect between emotional expression and context [*F*_(3,174)_ = 0.38, *p =* 0.769, ƞ_p_^2^ = 0.01, Ɛ = 0.96] or a three-way interaction [*F*_(3,174)_ = 0.10, *p =* 0.959, ƞ_p_^2^ = 0.002, Ɛ = 1].

**Figure 6:**
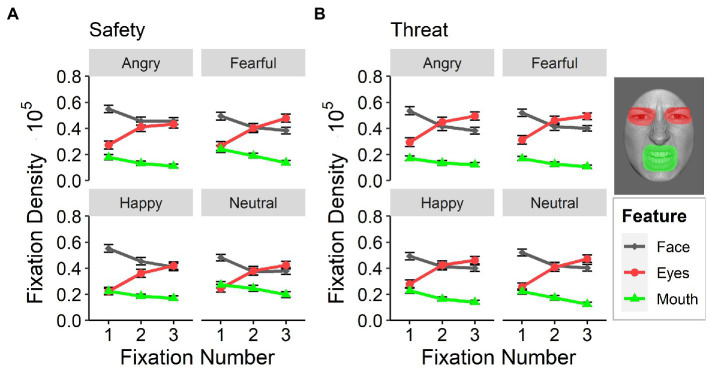
Sequential fixations. Fixation densities for the first three sequential fixations after stimulus onset as a function of emotional facial expression (angry, fearful, happy, and neutral) and attended feature (face, eyes, and mouth) for **(A)** the safety context and **(B)** the threat context. Error bars indicate standard errors of the mean.

With regard to the first three *sequential fixations*, the eyes of the presented facial expressions gained increased focus [main effect of feature: *F*_(2,118)_ = 34.85, *p <* 0.001, ƞ_p_^2^ = 0.37, Ɛ = 0.67], especially under threat [interaction effect between feature and context: *F*_(2,116)_ = 17.89, *p <* 0.001, ƞ_p_^2^ = 0.24, Ɛ = 0.98], (Figure 6). Participants successively prioritized the eye region [interaction between fixation number and feature: *F*_(4,232)_ = 37.09, *p <* 0.001, ƞ_p_^2^ = 0.31, Ɛ = 0.69], especially for fearful and neutral faces [interaction between feature and emotional facial expression: *F*_(6,348)_ = 7.79, *p <* 0.001, ƞ_p_^2^ = 0.12, Ɛ = 0.77]. The eyes of fearful faces showed the highest fixation density overall within the first three fixations, yet differences between threat and safety context were evident for all facial expressions (all *t* > 2.26, *p* < 0.025; paired). Differences between contexts in fixation densities for the mouth region were only evident for fearful [*t*_(179)_ = −5.09, *p* < 0.001, *d* = 0.37; paired] and neutral [*t*_(179)_ = −6.08, *p* < 0.001, *d* = 0.40; paired] facial expressions [3-way interaction between emotional facial expression, feature, and context: *F*_(6,348)_ = 4.19, *p =* 0.004, ƞ_p_^2^ = 0.07, Ɛ = 0.85], such that the overall fixation density for both expressions was higher in safety contexts than in threat contexts.

#### Influence of person-related variables

Descriptive statistics of the self-reported questionnaire data can be found in Table 1. Social anxiety traits correlated positively with the difference in accuracy (*r_s_* = 0.233*, p* = 0.015) and saccade onsets (*r_s_* = 0.230, *p* = 0.030) between safety and threat for fearful faces. Trait anxiety (STAI-T) scores negatively correlated with the difference in dwell times on fearful (*r_s_* = −0.307, *p* = 0.017) and neutral faces (*r_s_* = −0.325, *p* = 0.011) and positively correlated with the difference in d’ values for fearful faces (*r_s_* = 0.201, *p* = 0.030). State anxiety after the experiment correlated negatively with the difference in dwell time between safety and threat on fearful faces (*r_s_* = −0.275, *p* = 0.034). Interestingly, the sum score of the Autism Quotient Questionnaire negatively correlated with the accuracy of happy faces in the threat context (*r_s_* = −0.209, *p* = 0.024). Although this relationship was not observed in the safety context (*r_s_* = −0.092, *p* = 0.323), the difference between safety and threat for happy faces did not correlate with the sum score of the Autism Quotient Questionnaire to the same extent (*r_s_* = 0.164, *p* = 0.078). We did not find evidence that reaction times onto different features correlated with any other questionnaire measures.

**Table 1:** Psychometric measures across all participants depicting means (standard deviations) and ranges for each assessed questionnaire.

**Measure**	**Mean (*SD*)**	**Range**
Liebowitz Social Anxiety Scale	37.51 (19.81)	0–91
Autism Quotient (sum score)	6.98 (3.63)	0–17
*Social Interaction*	1.60 (1.80)	0–8
*Imagination*	2.36 (1.97)	0–7
*Communication*	2.93 (1.65)	0–9
LSRP (sum score)	46.03 (7.92)	30–76
*Interpersonal features*	27.51 (6.03)	19–46
*Social Deviance*	18.52 (2.71)	11–30
STAI-State (before experiment)	34.51 (5.88)	23–53
STAI-State (after experiment)	36.97 (6.58)	26–61
STAI-Trait	38.56 (8.69)	22–59
Positive Affect	20.56 (3.51)	12–35
Negative Affect	24.12 (4.37)	14–34

## Discussion

This study investigated differences for classifying emotions in two situational contexts by means of eye gaze and behavioral data. Specifically, our paradigm focused on contexts that are associated with social situations and their influence on interpreting emotional expressions and intentions of others.

On a behavioral level, our results showed that reaction times for categorizing emotions presented in a threatening context were slower than for those in a safety context, which is in line with our first hypothesis. Accuracy was lower in threat contexts overall, but threat particularly influenced the categorization of non-negative emotions. These expressions (happy and neutral) were misclassified as fearful more often under threat than in safety (see Figure 3), suggesting that threat applies a negative (or fearful) filter to the perception of neutral and positive information, for example by altering network weights between representations of such non-negative stimuli and representations of fear information within the cognitive architecture. With regard to gaze behavior, the data revealed a later onset of the first saccade upon stimulus presentation for faces presented in the threatening context, as well as more fixation changes in response to all emotional expressions. Dwell times were shorter under threat with negative emotions being attended the least. There was also a higher focus on the mouth region across all presented faces under threat and a higher focus on the eyes in safety contexts. Sequential fixations, however show, that the first three fixations are predominantly directed toward the eyes (especially of fearful faces), yet the eyes are subsequently avoided under threat, as indicated by overall dwell time. This pattern is similar to ones found in patients with social phobia (Boll et al., 2016). The new finding here is that the very first saccade initiation is delayed under threat and that this tendency is even evident in healthy participants in aversive anticipation. This study therefore indicates that responses under threat induction in healthy individuals resemble the behavior of individuals with social phobia in otherwise normal scenarios.

### Associations between threat and psychometric data

Generally, alterations in emotion processing are most evident in disorders that affect social functions like social phobia/anxiety and autism-spectrum disorders (Wieser and Brosch, 2012), which are also associated with decreased interpersonal emotion knowledge (e.g., Mennin et al., 2009). In the current study, higher autistic trait scores were associated with lower accuracy for happy faces in safety contexts. This is in contrast to findings in individuals on the autism spectrum, where emotion recognition impairments were mostly found for negative emotions (Ashwin et al., 2006; Farran et al., 2011). Higher social anxiety correlated with a delay in saccade initiation and an increase in accuracy between safety and threat contexts when fearful faces were presented. Such a finding is related to social anxiety being associated with enhanced detection of negative emotions (Gutiérrez-García and Calvo, 2017). Additionally, higher state and trait anxiety were related to shorter dwell times on fearful faces in our sample. Previous literature indicates longer dwell times for threatening faces (i.e., anger and fear) for individuals with high anxiety levels (Lazarov et al., 2016), which is why this gaze behavior was also expected for threatening contexts.

### Clinical implications

Our results suggest context-dependent behavioral and attentional changes in emotion recognition abilities in healthy subjects. Follow-up studies on patients with detected deficits in recognizing and processing emotional expressions, e.g., personality disorders, such as borderline personality disorder (for review see Domes et al., 2009), developmental disorders like autism-spectrum disorders (for review see Harms et al., 2010), and mental disorders, such as schizophrenia (for review see Edwards et al., 2002), major depressive disorder (for review see Bourke et al., 2010), and post-traumatic stress disorder (for review see Hayes et al., 2012) may yield aberrant patterns. Correlation results of our study may indicate specific emotion recognition deficits that are augmented under threat (e.g., higher Autistic traits correlate negatively with accuracy for happy faces only in the threat context). Particularly individuals with a negativity bias may show an even more conspicuous behavior toward fear under threat.

Recent studies investigating self-updating (Müller-Pinzler et al., 2019; Czekalla et al., 2021) and integration of social feedback (Korn et al., 2016a) have revealed interesting adaptational abilities in adulthood (see also review of Flechsenhar et al., 2022). As such, as Robinson et al. (2013b) suggested, some of the deficits anxious patients encounter may be secondary to, or occur in the context of, a poor ability to apply attentional resources (cognitive control) to flexibly adjust attention in the face of changing environments (Derryberry and Reed, 2002; Eysenck et al., 2007) and further also depend highly on environmental stressors (e.g., Bar-Haim et al., 2010). This indicates that state anxiety is a key variable in the modulation of bias (Robinson et al., 2013b). Individuals with borderline personality disorder have difficulties processing self-referential feedback in social interactions (Korn et al., 2016b) and seem to learn more slowly with a heightened sensitivity for environmental changes (Henco et al., 2020). Individuals diagnosed with schizophrenia also show impairments in context processing that have been associated with inferential components of social cognition in the disorder (Chung et al., 2011). Persons scoring high on the autism spectrum showed a reduced ability to implicitly encode and integrate contextual cues needed to access social meaning (Baez and Ibanez, 2014). Context dependency is therefore an important aspect to consider when defining social competencies, as well as their potential for adaptation. However, previous research on contextual differences has not focused on the flexibility to change across the experiment as a function of context or individual priors (for review see Flechsenhar et al., 2022).

### Theoretical implications

**Figure 7:**
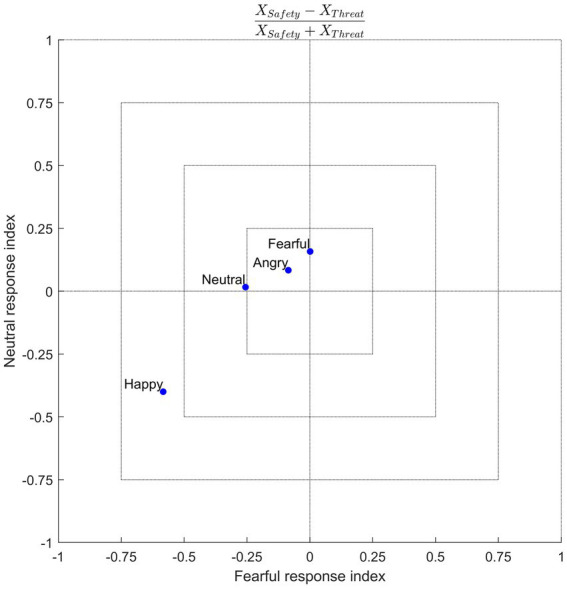
Conceptual illustration of response space. Axes reflect a normalized response index, calculated from the group-averaged confusion matrices as (e.g., for fearful responses) [Fearful_Safety_ − Fearful_Threat_]/[Fearful_Safety_ + Fearful _Threat_], that shows a response bias in the safety (positive values) or threat (negative values) contexts for each of the four emotion conditions (blue dots). Here one sees that fearful and neutral responses were more common on trials in which a happy face was presented in the threat context. This conceptualization demonstrates the extent to which the threat context selectively biases the response space for non-negative emotions.

Previous research into emotion recognition has generally applied context manipulations to ambiguous faces. Using unambiguous emotional expressions, the results of this study suggest that threat renders non-negative emotions to be perceived more negatively, such that, compared to a safe context, neutral faces tend to be misclassified as being fearful, while happy faces tend to be misclassified as more neutral and fearful. The fact that this effect is *not* uniform (i.e., misclassification of all other emotions as fearful, along with an increase in true positives for fearful expressions, under threat) helps to constrain theories regarding the nature of the underlying mechanisms involved in recognizing emotions, especially with respect to information processing in patients with negativity biases (or general emotion recognition deficits). One speculation is that threat biases decision-making processes toward a negative state. This would suggest that the abstract representation of emotions compresses, warping “positive” stimuli toward the “negative” side of this representational space. Another speculation is that of context-emotion congruency, which would suggest that the induction of a fearful context augments the tendency to classify facial expressions as fearful, as was the case in this study (albeit still with a positive-to-negative bias). Further examination is needed to confirm this notion, for instance, by testing other aversive context manipulations, such as disgust (Aviezer et al., 2008), or even context manipulations using appetitive stimuli, such as pleasant touch (e.g., Ellingsen et al., 2013; Kryklywy et al., 2021). The study by Schulreich et al. (2020) supports a context-dependent involvement of valuation processes, as they found fear-induced shifts from positive to negative value coding. Figure 7 offers a conceptual illustration of responses given in the current study. This data-driven visualization indicates a context-congruent shift to bias responses negatively (toward fear) for non-negative emotions.

### Related research

Related research has recently investigated differences in emotion information processing using behavioral techniques. For example, threat-of-shock leads to a negative bias in categorizing ambiguous faces (Neta et al., 2017), patients with borderline personality disorder appear to show a negative bias in judging facial expressions (Fenske et al., 2015) and ambiguous emotional expressions (Kleindienst et al., 2019), and healthy individuals with high neuroticism and low conscientiousness tend to differentiate negatively valenced information to a greater degree (Levine et al., 2020). Recent neuroimaging research has also explored various factors that contribute to altered affective information processing the brain. For example, aversive-learning renders activity patterns of semantically distinct categories more similar in anterior temporal and superior frontal regions (Levine et al., 2018, 2021), oxytocin attenuates a stress-induced emotion recognition bias in anterior temporal regions and anterior cingulate cortex (Maier et al., 2019), and cerebellar activity in patients with major depressive disorder modulates a negativity bias in emotion recognition (Nakamura et al., 2022). These findings, in combination with the present results, raise the question of whether factors such as threat, personality, and mood (*inter alia*) involve common cognitive systems or altered representational spaces (and their neurobiological correlates) that play a more general role in affective processing.

### Limitations

The utilized emotion recognition paradigm, although validated and applied in several other studies (e.g., Scheller et al., 2012; Boll et al., 2016; Bertsch et al., 2017), directly immerses participants into the displayed emotional faces, which minimizes the voluntary and nuanced analysis of attentional deployment. Further, deviating from other attentional choice tasks, our paradigm only presents the participant with one face that they can choose to focus on and explore. However, the fact that the present eye tracking results nevertheless depict attentional changes is in line with previous work.

## Conclusion

To understand fundamental social processing as a function of situational influence, we aimed to characterize the influence of threat on emotion recognition in healthy individuals. The results confirmed that emotion recognition is influenced by threat across behavioral and eye tracking measures. Future research should continue investigating the influence of context on social perception and interactions, especially for patients with mental or personality disorders. Underlying processes could be addressed to find the cause of shifting from adaptive to maladaptive anxiety and follow-up on flexible cognitive adaptation abilities within social cognition processes to improve social skills for patients experiencing social dysfunction. Further application of paradigms incorporating different influencing factors, such as context and allowing for behavioral changes over the course of the experiment, as well as a stronger focus on behavior in actual social interactions (e.g., Abramson et al., 2021), may indicate a large-scale rigidity that contributes to symptomatology of social deficits and may help uncover whether maladaptive adaptation mechanisms contribute to social dysfunctions as part of their symptomatology. Understanding threat processing, systematic changes in representational spaces, and altered decision thresholds may additionally inform psychotherapeutic and pharmacological interventions.

## Data availability statement

The datasets presented in this study can be found in online repositories. The names of the repository/repositories and accession number(s) can be found at: Open Science Framework: https://osf.io/x6ucv/?view_only=0312777950e7487a89c7b0e8bd11b6ab.

## Ethics statement

The studies involving human participants were reviewed and approved by Ethics Committee of the Ludwig-Maximilians Universität München, Department Psychology and Education. The patients/participants provided their written informed consent to participate in this study.

## Author contributions

AF and KB: conceptualization, methodology. AF: data curation, eye tracking analysis, drafting manuscript. AF and SL: behavioral analysis, data visualization. AF, SL, and KB: writing, revising, and finalizing manuscript. KB: experiment resources. All authors contributed to the article and approved the submitted version.

## Conflict of interest

The authors declare that the research was conducted in the absence of any commercial or financial relationships that could be construed as a potential conflict of interest.

## Publisher’s note

All claims expressed in this article are solely those of the authors and do not necessarily represent those of their affiliated organizations, or those of the publisher, the editors and the reviewers. Any product that may be evaluated in this article, or claim that may be made by its manufacturer, is not guaranteed or endorsed by the publisher.

## Supplementary material

The Supplementary material for this article can be found online at: https://www.frontiersin.org/articles/10.3389/fpsyg.2022.967800/full#supplementary-material

## Footnotes

^1^ www.r-project.org

## References

[ref1] Abramson L. Petranker R. Marom I. Aviezer H. (2021). Social interaction context shapes emotion recognition through body language, not facial expressions. Emotion 21, 557–568. doi: 10.1037/emo0000718, PMID: 31971411

[ref1003] Alpers G. W. Gerdes A. B. M. (2007). Here is looking at you: Emotional faces predominate in binocular rivalry. Emotion 7, 495–506. doi: 10.1037/1528-3542.7.3.495 17683206

[ref2] Ashwin C. Chapman E. Colle L. Baron-Cohen S. (2006). Impaired recognition of negative basic emotions in autism: a test of the amygdala theory. Soc. Neurosci. 1, 349–363. doi: 10.1080/17470910601040772, PMID: 18633799

[ref3] Asmundson G. J. Stein M. B. (1994). Selective processing of social threat in patients with generalized social phobia: evaluation using a dot-probe paradigm. J. Anxiety Disord. 8, 107–117. doi: 10.1016/0887-6185(94)90009-4

[ref4] Aviezer H. Hassin R. R. Ryan J. Grady C. Susskind J. Anderson A.. (2008). Angry, disgusted, or afraid? Psychol. Sci. 19, 724–732. doi: 10.1111/j.1467-9280.2008.02148.x 18727789

[ref5] Baddeley A. Rubak E. Turner R. (2015). Spatial point patterns: Methodology and applications with R. Chapman and Hall/CRC Press. Available at: http://www.crcpress.com/Spatial-Point-Patterns-Methodology-and-Applications-with-R/Baddeley-Rubak-Turner/9781482210200/

[ref6] Baez S. Ibanez A. (2014). The effects of context processing on social cognition impairments in adults with Asperger’s syndrome. Front. Neurosci. 8:270. doi: 10.3389/fnins.2014.00270, PMID: 25232301 PMC4153041

[ref7] Bakeman R. (2005). Recommended effect size statistics for repeated measures designs. Behav. Res. Methods 37, 379–384. doi: 10.3758/BF03192707, PMID: 16405133

[ref8] Baker S. L. Heinrichs N. Kim H.-J. Hofmann S. G. (2002). The Liebowitz social anxiety scale as a self-report instrument: a preliminary psychometric analysis. Behav. Res. Ther. 40, 701–715. doi: 10.1016/S0005-7967(01)00060-2, PMID: 12051488

[ref9] Bar-Haim Y. Holoshitz Y. Eldar S. Frenkel T. I. Muller D. Charney D. S.. (2010). Life-threatening danger and suppression of attention bias to threat. Am. J. Psychiatr. 167, 694–698. doi: 10.1176/appi.ajp.2009.09070956, PMID: 20395400

[ref10] Bertsch K. Krauch M. Stopfer K. Haeussler K. Herpertz S. C. Gamer M. (2017). Interpersonal threat sensitivity in borderline personality disorder: an eye-tracking study. J. Personal. Disord. 31, 647–670. doi: 10.1521/pedi_2017_31_273, PMID: 28072041

[ref11] Black M. H. Chen N. T. Lipp O. V. Bölte S. Girdler S. (2020). Complex facial emotion recognition and atypical gaze patterns in autistic adults. Autism 24, 258–262. doi: 10.1177/1362361319856969, PMID: 31216863

[ref12] Boll S. Bartholomaeus M. Peter U. Lupke U. Gamer M. (2016). Attentional mechanisms of social perception are biased in social phobia. J. Anxiety Disord. 40, 83–93. doi: 10.1016/j.janxdis.2016.04.004, PMID: 27131909 PMC4877390

[ref13] Bosse T. Schnitfink K. (2015). The effect of simulated threat on task performance during emotion recognition, Springer, Cham. 107–116.

[ref14] Bourke C. Douglas K. Porter R. (2010). Processing of facial emotion expression in major depression: a review. Australian & New Zealand J. Psychiatry 44, 681–696. doi: 10.3109/00048674.2010.496359, PMID: 20636189

[ref15] Bradley M. M. Lang P. J. (2007). “The international affective digitized sounds (2nd edition, IADS-2): affective ratings of sounds and instruction manual” in Technical report B-3 Oxford University Press.

[ref16] Brinkley C. A. Schmitt W. A. Smith S. S. Newman J. P. (2001). Construct validation of a self-report psychopathy scale: does Levenson’s self-report psychopathy scale measure the same constructs as Hare’s psychopathy checklist-revised? Personal. Individ. Differ. 31, 1021–1038. doi: 10.1016/S0191-8869(00)00178-1

[ref17] Bublatzky F. Gerdes A. B. M. White A. J. Riemer M. Alpers G. W. (2014). Social and emotional relevance in face processing: happy faces of future interaction partners enhance the late positive potential. Front. Hum. Neurosci. 8:493. doi: 10.3389/fnhum.2014.00493, PMID: 25076881 PMC4100576

[ref18] Bublatzky F. Kavcıoğlu F. Guerra P. Doll S. Junghöfer M. (2020). Contextual information resolves uncertainty about ambiguous facial emotions: behavioral and magnetoencephalographic correlates. NeuroImage 215:116814. doi: 10.1016/j.neuroimage.2020.116814, PMID: 32276073

[ref19] Calvo M. G. Fernández-Martín A. Nummenmaa L. (2014). Facial expression recognition in peripheral versus central vision: role of the eyes and the mouth. Psychol. Res. 78, 180–195. doi: 10.1007/s00426-013-0492-x, PMID: 23595965

[ref20] Chung Y. S. Mathews J. R. Barch D. M. (2011). The effect of context processing on different aspects of social cognition in schizophrenia. Schizophr. Bull. 37, 1048–1056. doi: 10.1093/schbul/sbq012, PMID: 20185539 PMC3160231

[ref21] Cisler J. M. Koster E. H. W. (2010). Mechanisms of attentional biases towards threat in anxiety disorders: an integrative review. Clin. Psychol. Rev. 30, 203–216. doi: 10.1016/j.cpr.2009.11.003, PMID: 20005616 PMC2814889

[ref22] Cohen J. (1962). The statistical power of abnormal-social psychological research: a review. J. Abnorm. Soc. Psychol. 65, 145–153. doi: 10.1037/h0045186, PMID: 13880271

[ref23] Crawford J. R. Henry J. D. (2004). The positive and negative affect schedule (PANAS): construct validity, measurement properties and normative data in a large non-clinical sample. Br. J. Clin. Psychol. 43, 245–265. doi: 10.1348/0144665031752934 15333231

[ref24] Crouzet S. M. (2010). Fast saccades toward faces: face detection in just 100 ms. J. Vis. 10, 1–17. doi: 10.1167/10.4.16, PMID: 20465335

[ref25] Czekalla N. Stierand J. Stolz D. S. Mayer A. V. Voges J. F. Rademacher L.. (2021). Self-beneficial belief updating as a coping mechanism for stress-induced negative affect. Sci. Rep. 11, 17096–17013. doi: 10.1038/s41598-021-96264-0, PMID: 34429447 PMC8384941

[ref26] de Gelder B. Vroomen J. (2000). Rejoinder-bimodal emotion perception: integration across separate modalities, cross-modal perceptual grouping or perception of multimodal events? Cognit. Emot. 14, 321–324. doi: 10.1080/026999300378842

[ref27] Derryberry D. Reed M. A. (2002). Anxiety-related attentional biases and their regulation by attentional control. J. Abnorm. Psychol. 111, 225–236. doi: 10.1037/0021-843X.111.2.225, PMID: 12003445

[ref28] Diéguez-Risco T. Aguado L. Albert J. Hinojosa J. A. (2013). Faces in context: modulation of expression processing by situational information. Soc. Neurosci. 8, 601–620. doi: 10.1080/17470919.2013.834842, PMID: 24053118

[ref29] Diéguez-Risco T. Aguado L. Albert J. Hinojosa J. A. (2015). Judging emotional congruency: explicit attention to situational context modulates processing of facial expressions of emotion. Biol. Psychol. 112, 27–38. doi: 10.1016/j.biopsycho.2015.09.012, PMID: 26450006

[ref30] Domes G. Schulze L. Herpertz S. C. (2009). Emotion recognition in borderline personality disorder—a review of the literature. J. Personal. Disord. 23, 6–19. doi: 10.1521/pedi.2009.23.1.6, PMID: 19267658

[ref31] Ebner N. C. Riediger M. Lindenberger U. (2010). FACES—A database of facial expressions in young, middle-aged, and older women and men: development and validation. Behav. Res. Methods 42, 351–362. doi: 10.3758/BRM.42.1.351, PMID: 20160315

[ref32] Edwards J. Jackson H. J. Pattison P. E. (2002). Emotion recognition via facial expression and affective prosody in schizophrenia. Clin. Psychol. Rev. 22, 789–832. doi: 10.1016/S0272-7358(02)00130-7, PMID: 12214327

[ref33] Ellingsen D.-M. Wessberg J. Eikemo M. Liljencrantz J. Endestad T. Olausson H.. (2013). Placebo improves pleasure and pain through opposite modulation of sensory processing. Proc. Natl. Acad. Sci. 110, 17993–17998. doi: 10.1073/pnas.1305050110, PMID: 24127578 PMC3816412

[ref34] Eysenck M. W. Derakshan N. Santos R. Calvo M. G. (2007). Anxiety and cognitive performance: attentional control theory. Emotion 7, 336–353. doi: 10.1037/1528-3542.7.2.336 17516812

[ref35] Farran E. K. Branson A. King B. J. (2011). Visual search for basic emotional expressions in autism; impaired processing of anger, fear and sadness, but a typical happy face advantage. Res. Autism Spectr. Disord. 5, 455–462. doi: 10.1016/j.rasd.2010.06.009

[ref36] Faul F. Erdfelder E. Lang A.-G. Buchner A. (2007). GPOWER: a general power analysis program. Behav. Res. Methods 39, 175–191. doi: 10.3758/BF03193146, PMID: 17695343

[ref37] Fenske S. Lis S. Liebke L. Niedtfeld I. Kirsch P. Mier D. (2015). Emotion recognition in borderline personality disorder: effects of emotional information on negative bias. Borderline Personality Disorder and Emotion Dysregulation 2:10. doi: 10.1186/s40479-015-0031-z, PMID: 26401312 PMC4579484

[ref38] Flechsenhar A. Gamer M. (2017). Top-down influence on gaze patterns in the presence of social features. PLoS One 12:e0183799. doi: 10.1371/journal.pone.0183799, PMID: 28837673 PMC5570331

[ref39] Flechsenhar A. Kanske P. Krach S. Korn C. Bertsch K. (2022). The (un)learning of social functions and its significance for mental health. Clin. Psychol. Rev. 98:102204. doi: 10.1016/j.cpr.2022.102204, PMID: 36216722

[ref40] Flechsenhar A. Larson O. End A. Gamer M. (2018a). Investigating overt and covert shifts of attention within social naturalistic scenes. J. Vis. 18:11. doi: 10.1167/18.12.11, PMID: 30458516

[ref41] Flechsenhar A. Rösler L. Gamer M. (2018b). Attentional selection of social features persists despite restricted bottom-up information and affects temporal viewing dynamics. Sci. Rep. 8:12555. doi: 10.1038/s41598-018-30736-8, PMID: 30135443 PMC6105690

[ref42] Freitag C. M. Retz-Junginger P. Retz W. Seitz C. Palmason H. Meyer J.. (2007). Evaluation der deutschen Version des Autismus-Spektrum-Quotienten (AQ) - Die kurzversion AQ-k. Zeitschrift Fur Klinische Psychologie Und Psychotherapie 36, 280–289. doi: 10.1026/1616-3443.36.4.280

[ref1001] Gray J. A. (2019). Issues in the neuropsychology of anxiety. In Anxiety and the anxiety disorders. Routledge. 5–26.

[ref43] Gregory B. Wong Q. J. J. Marker C. D. Peters L. (2018). Maladaptive self-beliefs during cognitive Behavioural therapy for social anxiety disorder: a test of temporal precedence. Cogn. Ther. Res. 42, 261–272. doi: 10.1007/s10608-017-9882-5

[ref44] Gutiérrez-García A. Calvo M. G. (2017). Social anxiety and threat-related interpretation of dynamic facial expressions: sensitivity and response bias. Personal. Individ. Differ. 107, 10–16. doi: 10.1016/j.paid.2016.11.025

[ref45] Harms M. B. Martin A. Wallace G. L. (2010). Facial emotion recognition in autism Spectrum disorders: a review of behavioral and neuroimaging studies. Neuropsychol. Rev. 20, 290–322. doi: 10.1007/s11065-010-9138-6, PMID: 20809200

[ref46] Hayes J. P. VanElzakker M. B. Shin L. M. (2012). Emotion and cognition interactions in PTSD: a review of neurocognitive and neuroimaging studies. Front. Integr. Neurosci. 6:89. doi: 10.3389/fnint.2012.00089, PMID: 23087624 PMC3466464

[ref47] Heimberg R. G. Horner K. J. Juster H. R. Safren S. A. Brown E. J. Schneier F. R.. (1999). Psychometric properties of the Liebowitz social anxiety scale. Psychol. Med. 29, 199–212. doi: 10.1017/S0033291798007879 10077308

[ref48] Henco L. Diaconescu A. O. Lahnakoski J. M. Brandi M.-L. Hörmann S. Hennings J.. (2020). Aberrant computational mechanisms of social learning and decision-making in schizophrenia and borderline personality disorder. PLoS Comput. Biol. 16:e1008162. doi: 10.1371/journal.pcbi.1008162, PMID: 32997653 PMC7588082

[ref49] Heuer K. Rinck M. Becker E. S. (2007). Avoidance of emotional facial expressions in social anxiety: the approach–avoidance task. Behav. Res. Ther. 45, 2990–3001. doi: 10.1016/j.brat.2007.08.010 17889827

[ref50] Hicklin J. Widiger T. A. (2005). Similarities and differences among antisocial and psychopathic self-report inventories from the perspective of general personality functioning. Eur. J. Personal. 19, 325–342. doi: 10.1002/per.562

[ref51] Hietanen J. K. Leppänen J. M. (2008). Judgment of other people’s facial expressions of emotions is influenced by their concurrent affective hand movements. Scand. J. Psychol. 49, 221–230. doi: 10.1111/j.1467-9450.2008.00644.x, PMID: 18384495

[ref52] Huynh H. Feldt L. S. (1976). Estimation of the box correction for degrees of freedom from sample data in randomized block and Split-plot designs. J. Educ. Stat. 1, 69–82. doi: 10.3102/10769986001001069

[ref53] Janke S. Glöckner-Rist A. (2014). “Deutsche version der positive and negative affect schedule (PANAS)” in In Zusammenstellung sozialwissenschaftlicher Items und Skalen (Germany: GESIS), 6102.

[ref54] Kavcıoğlu F. C. Bublatzky F. Pittig A. Alpers G. W. (2021). Instructed threat enhances threat perception in faces. Emotion 21, 419–429. doi: 10.1037/emo0000708, PMID: 31829719

[ref55] Kimonis E. R. Kidd J. Most S. B. Krynen A. Liu C. (2020). An elusive deficit: psychopathic personality traits and aberrant attention to emotional stimuli. Emotion 20, 951–964. doi: 10.1037/emo0000601, PMID: 30945889

[ref1002] Kleindienst N. Hauschild S. Liebke L. (2019). A negative bias in decoding positive social cues characterizes emotion processing in patients with symptom-remitted Borderline Personality Disorder. Bord personal disord emot dysregul 6, 17. doi: 10.1186/s40479-019-0114-3 PMC6858731 31788316

[ref56] Korn C. W. La Rosée L. Heekeren H. R. Roepke S. (2016a). Social feedback processing in borderline personality disorder. Psychol. Med. 46, 575–587. doi: 10.1017/S003329171500207X, PMID: 26467724

[ref57] Korn C. W. Rosenblau G. Rodriguez Buritica J. M. Heekeren H. R. (2016b). Performance feedback processing is positively biased as predicted by attribution theory. PLoS One 11:e0148581. doi: 10.1371/journal.pone.0148581, PMID: 26849646 PMC4743912

[ref58] Kryklywy J. H. Ehlers M. R. Beukers A. O. Moore S. R. Todd R. M. Anderson A. K. (2021). Decoding representations of discriminatory and hedonic information during appetitive and aversive touch. BioRxiv Preprint 2020:383. doi: 10.1101/2020.09.24.310383

[ref59] Lang P. J. Bradley M. M. Cuthbert B. N. (2008). “International affective picture system (IAPS): affective ratings of pictures and instruction manual,” in Technical report. *Vol. A-8*, University of Florida, Gainesville, FL.

[ref60] Laux L. Glanzmann P. Schaffner P. Spielberger C. (1981). STAI: State-trait-Angstinventar, Weinheim: Beltz Test GmbH.

[ref61] Lawrence M. A. (2016). Ez: Easy analysis and visualization of factorial experiments.

[ref62] Lazarov A. Abend R. Bar-Haim Y. (2016). Social anxiety is related to increased dwell time on socially threatening faces. J. Affect. Disord. 193, 282–288. doi: 10.1016/j.jad.2016.01.007, PMID: 26774515

[ref63] Levenson M. R. Kiehl K. A. Fitzpatrick C. M. (1995). Assessing psychopathic attributes in a noninstitutionalized population. J. Pers. Soc. Psychol. 68, 151–158. doi: 10.1037/0022-3514.68.1.151, PMID: 7861311

[ref64] Levine S. M. Alahäivälä A. L. I. Wechsler T. F. Wackerle A. Rupprecht R. Schwarzbach J. V. (2020). Linking personality traits to individual differences in affective spaces. Front. Psychol. 11:448. doi: 10.3389/fpsyg.2020.00448, PMID: 32231631 PMC7082752

[ref65] Levine S. M. Kumpf M. Rupprecht R. Schwarzbach J. V. (2021). Supracategorical fear information revealed by aversively conditioning multiple categories. Cogn. Neurosci. 12, 28–39. doi: 10.1080/17588928.2020.1839039, PMID: 33135598

[ref66] Levine S. M. Pfaller M. Reichenberger J. Shiban Y. Mühlberger A. Rupprecht R.. (2018). Relating experimentally-induced fear to pre-existing phobic fear in the human brain. Soc. Cogn. Affect. Neurosci. 13, 164–172. doi: 10.1093/scan/nsx147, PMID: 29281096 PMC5827344

[ref67] Lilienfeld S. O. Fowler K. A. (2006). The self-report assessment of psychopathy: Problems, pitfalls, and promises. New York: Guilford Press.

[ref68] Lisk S. Vaswani A. Linetzky M. Bar-Haim Y. Lau J. Y. F. (2020). Systematic review and meta-analysis: eye-tracking of attention to threat in child and adolescent anxiety. J. Am. Acad. Child Adolesc. Psychiatry 59, 88–99. doi: 10.1016/j.jaac.2019.06.006, PMID: 31265874

[ref69] Lundqvist D. E. Flykt A. Öhman A. (1998). The Karolinska directed emotional faces-KDEF, CD ROM from Department of Clinical Neuroscience, Psychology section. Karolinska Institutet, Sweden.

[ref70] Lynam D. R. Whiteside S. Jones S. (1999). Self-reported psychopathy: a validation study. J. Pers. Assess. 73, 110–132. doi: 10.1207/S15327752JPA730108 10497804

[ref71] Macmillan N. A. Creelman C. D. (2004). Detection theory. London: Psychology Press.

[ref72] Maier A. Scheele D. Spengler F. B. Menba T. Mohr F. Güntürkün O.. (2019). Oxytocin reduces a chemosensory-induced stress bias in social perception. Neuropsychopharmacology 44, 281–288. doi: 10.1038/s41386-018-0063-3, PMID: 29703998 PMC6300531

[ref73] Mennin D. S. McLaughlin K. A. Flanagan T. J. (2009). Emotion regulation deficits in generalized anxiety disorder, social anxiety disorder, and their co-occurrence. J. Anxiety Disord. 23, 866–871. doi: 10.1016/j.janxdis.2009.04.006, PMID: 19464142 PMC4180233

[ref74] Miller J. D. Lyman D. R. Widiger T. A. Leukefeld C. (2001). Personality disorders as extreme variants of common personality dimensions: can the five factor model adequately represent psychopathy? J. Pers. 69, 253–276. doi: 10.1111/1467-6494.00144, PMID: 11339798

[ref75] Müller-Pinzler L. Czekalla N. Mayer A. V. Stolz D. S. Gazzola V. Keysers C.. (2019). Negativity-bias in forming beliefs about own abilities. Sci. Rep. 9, 14416–14415. doi: 10.1038/s41598-019-50821-w, PMID: 31594967 PMC6783436

[ref76] Nakamura A. Yomogida Y. Ota M. Matsuo J. Ishida I. Hidese S.. (2022). The cerebellum as a moderator of negative bias of facial expression processing in depressive patients. J. Affective Disorders Reports 7:100295. doi: 10.1016/j.jadr.2021.100295

[ref77] Neta M. Cantelon J. Haga Z. Mahoney C. R. Taylor H. A. Davis F. C. (2017). The impact of uncertain threat on affective bias: individual differences in response to ambiguity. Emotion 17, 1137–1143. doi: 10.1037/emo0000349, PMID: 28910121

[ref78] Osumi T. Ohira H. (2017). Selective fair behavior as a function of psychopathic traits in a subclinical population. Front. Psychol. 8:1604. doi: 10.3389/fpsyg.2017.01604, PMID: 28955290 PMC5601322

[ref79] Paret C. Bublatzky F. (2019). Threat rapidly disrupts reward reversal learning. Psyarvix 810. doi: 10.1016/j.brat.2020.103636 32387886

[ref80] Pourtois G. (2004). Electrophysiological correlates of rapid spatial orienting towards fearful faces. Cereb. Cortex 14, 619–633. doi: 10.1093/cercor/bhh023, PMID: 15054077

[ref81] R Core Team. (2016). R: A language and environment for statistical computing (3.2.3). R Foundation for Statistical Computing. Available at: https://www.r-project.org/

[ref82] Robinson O. J. Krimsky M. Grillon C. (2013a). The impact of induced anxiety on response inhibition. Front. Hum. Neurosci. 7:69. doi: 10.3389/fnhum.2013.00069, PMID: 23471118 PMC3590569

[ref83] Robinson O. J. Vytal K. Cornwell B. R. Grillon C. (2013b). The impact of anxiety upon cognition: perspectives from human threat of shock studies. Front. Hum. Neurosci. 7:203. doi: 10.3389/fnhum.2013.00203, PMID: 23730279 PMC3656338

[ref84] Roelofs K. van Peer J. Berretty E. de Jong P. Spinhoven P. Elzinga B. M. (2009). Hypothalamus–pituitary–adrenal Axis Hyperresponsiveness is associated with increased social avoidance behavior in social phobia. Biol. Psychiatry 65, 336–343. doi: 10.1016/j.biopsych.2008.08.022, PMID: 18947821

[ref85] Rösler L. Gamer M. (2019). Freezing of gaze during action preparation under threat imminence. Sci. Rep. 9:17215. doi: 10.1038/s41598-019-53683-4, PMID: 31748589 PMC6868270

[ref86] Ross S. R. Lutz C. J. Bailley S. E. (2004). Psychopathy and the five factor model in a noninstitutionalized sample: a domain and facet level analysis. J. Psychopathol. Behav. Assess. 26, 213–223. doi: 10.1023/B:JOBA.0000045337.48535.a5

[ref87] Scheller E. Büchel C. Gamer M. (2012). Diagnostic features of emotional expressions are processed preferentially. PLoS One 7:e41792. doi: 10.1371/journal.pone.0041792, PMID: 22848607 PMC3405011

[ref88] Schmid P. C. Schmid Mast M. (2010). Mood effects on emotion recognition. Motiv. Emot. 34, 288–292. doi: 10.1007/s11031-010-9170-0

[ref89] Schmid P. C. Schmid Mast M. Bombari D. Mast F. W. Lobmaier J. S. (2011). How mood states affect information processing during facial emotion recognition: an eye tracking study. Swiss J. Psychol. 70, 223–231. doi: 10.1024/1421-0185/a000060

[ref90] Schulreich S. Gerhardt H. Meshi D. Heekeren H. R. (2020). Fear-induced increases in loss aversion are linked to increased neural negative-value coding. Soc. Cogn. Affect. Neurosci. 15, 661–670. doi: 10.1093/scan/nsaa091, PMID: 32644143 PMC7438956

[ref91] Seitz K. I. Leitenstorfer J. Krauch M. Hillmann K. Boll S. Ueltzhoeffer K.. (2021). An eye-tracking study of interpersonal threat sensitivity and adverse childhood experiences in borderline personality disorder. Borderline Personality Disorder and Emotion Dysregulation 8, 1–12. doi: 10.1186/s40479-020-00141-7 33397512 PMC7784013

[ref92] Sheppes G. Luria R. Fukuda K. Gross J. J. (2013). There’s more to anxiety than meets the eye: isolating threat-related attentional engagement and disengagement biases. Emotion 13, 520–528. doi: 10.1037/a0031236, PMID: 23356563

[ref93] Stangier U. Heidenreich T. (2005). “Liebowitz social anxiety scale” in Internationale Skalen für Psychiatrie (Internatioal scales for psychiatry) (Germany: Beltz), 299–306.

[ref94] Tottenham N. Tanaka J. W. Leon A. C. McCarry T. Nurse M. Hare T. A.. (2009). The NimStim set of facial expressions: judgments from untrained research participants. Psychiatry Res. 168, 242–249. doi: 10.1016/j.psychres.2008.05.006, PMID: 19564050 PMC3474329

[ref95] Watson D. Clark L. A. Tellegen A. (1988). Development and validation of brief measures of positive and negative affect: the PANAS scales. J. Pers. Soc. Psychol. 54, 1063–1070. doi: 10.1037/0022-3514.54.6.1063, PMID: 3397865

[ref96] Wieser M. J. Brosch T. (2012). Faces in context: a review and systematization of contextual influences on affective face processing. Front. Psychol. 3:471. doi: 10.3389/fpsyg.2012.00471, PMID: 23130011 PMC3487423

[ref97] World Health Organization (2020). International statistical classification of diseases and related health problems (*11th Edn.*). Available at: https://icd.who.int 10.2471/BLT.25.294650 PMC13531092 42683092

