# Supplementary material for: Threat induction biases processing of emotional expressions
Source: Front Psychol. 2022 Nov 24;13:967800. doi: 10.3389/fpsyg.2022.967800 (PMC9730731; doi:10.3389/fpsyg.2022.967800)
Supplement: Supplementary file 1 [file Data_Sheet_1.docx]

**Supplementary Figures**

Safety

| Target | Angry | 90.27 | 7.13 | 0.18 | 2.42 |
| --- | --- | --- | --- | --- | --- |
|  | Fearful | 6.19 | 91.95 | 0.51 | 1.36 |
|  | Happy | 0.41 | 0.29 | 98.79 | 0.51 |
|  | Neutral | 2.58 | 2.78 | 0.40 | 94.24 |
|  |  | Angry | Fearful | Happy | Neutral |
|  |  | Response | | | |

****Threat

| Target | Angry | 89.36 | 8.50 | 0.233 | 1.92 |
| --- | --- | --- | --- | --- | --- |
|  | Fearful | 6.12 | 92.58 | 0.28 | 1.02 |
|  | Happy | 0.40 | 1.08 | 97.33 | 1.19 |
|  | Neutral | 3.31 | 4.72 | 0.80 | 91.18 |
|  |  | Angry | Fearful | Happy | Neutral |
|  |  | Response | | | |

**Figure S1.** Confusion matrices for each context with the respective group-averaged responses (relative frequencies) given by the participants (columns) for each target emotional expression (rows). On the left are the confusion matrices as heat maps depicting absolute frequencies from 15 potential trials.

**Table S1.** Measures for reaction times, accuracy, saccades and fixations for each context across the four emotional expressions (angry, fearful, happy, neutral) for each experimental setup presenting the stimuli either for 150 ms (study 1) or 5000 ms (study 2), with the exception of dwell times, which can only be measured over longer periods of time.

*Note: NA refers to saccade onsets outside the criteria or presentation time. There was either no initiation of the first saccade at all within 150 ms, or the measures were below 50 ms. The latter does not allow us to draw conclusions on stimulus-specific reflexive eye movements.*
